# Clinical research training

**DOI:** 10.1590/1516-3180.2014.1324887

**Published:** 2014-05-20

**Authors:** Alessandro Wasum Mariani, Paulo Manuel Pêgo-Fernandes

**Affiliations:** I MD. Thoracic Surgeon, Instituto do Coração (InCor), Hospital das Clínicas (HC), Faculdade de Medicina da Universidade de São Paulo (FMUSP), São Paulo, Brazil; II MD, PhD. Associate Professor, Discipline of Thoracic Surgery, Instituto do Coração (InCor), Hospital das Clínicas (HC), Faculdade de Medicina da Universidade de São Paulo (FMUSP), São Paulo, Brazil

Scientific clinical research is a complex activity nowadays. Intricate regulatory issues, the need to raise funding, ethical issues and the growing difficulty in getting space for publication in high-impact journals mean that clinical research is no longer a place for amateurs. Many players within this scenario have already understood this new reality. Many of them consider that the 1990s was the decade when the clinical research environment dramatically changed, especially in the United States, through the addition of federal regulations and evolution in academic medicine.^1^ One natural consequence of this movement has been the emergence of clinical research courses. Our objective in this editorial was to show some examples of this new branch of academic and scientific activity, i.e. educational training for clinical researchers.

*Strictu sensu* postgraduate programs (mainly at doctoral level) are no longer the only way to get formal training for research. Many authors believe that this is also no longer the best way to achieve complete training because of the academic specificities of such programs. The pressure to acquire adequate training is largely generated by the increasing labor market relating to clinical research. In order to sponsor trials, the big companies require specific training for clinical research, especially for the main researchers, reviewers and auditors involved in the trial.

Several of the greatest universities in the world have specific training programs for clinical research. Example of these include Duke University (USA), McMaster University (Canada), University of Sheffield (UK) and Kyoto University (Japan), among many others.

Harvard Medical School has a successful six-month collaborative distance-learning basic course program called "Principles and Practice of Clinical Research", which has been taken by significant numbers of students worldwide. In 2012, the course was run through 13 international sites, with students from 25 countries, reaching more than 300 participants. This course covers the basics of clinical research (how to formulate a research question, select a study population and use randomization and blinding methods), statistical methods, data gathering, monitoring, reporting and study designs.^2^

In Brazil, our example is the online course promoted by the Discipline of Telemedicine of the University of São Paulo Medical School (Faculdade de Medicina da Universidade de São Paulo, FMUSP) called "*Princípios de Pesquisa Clínica*", which also has the objective of transmitting clinical research knowledge to aspiring researchers.^3^ It is a course intended not only for physicians but also for multidisciplinary healthcare team members like nurses, physiotherapists, speech therapists, etc. It should be borne in mind that telemedicine and teleducation as forms of distance learning are valuable tools in a country like Brazil with continental dimensions.

Many of these educational initiatives require payment of fees. Such courses are beginning to represent an important source of income for institutions and for people involved with this new type of specialized education. However, free options also exist, including online courses.^4^

Other ways to get this kind of training include the frequent opportunities offered by companies that are interested in training researchers among their own staff. Pharmaceutical companies are the major example; however, many healthcare institutions like hospitals are also looking into clinical research training with great interest.

Some of these training programs are complete courses including all aspects of clinical research, from ethical issues to methodology, and even go into how to raise funds and how to write scientific reports. Others aim to teach specific aspects of clinical research, like statistics courses or scientific methodology courses. 

We conclude that formal training to perform clinical research is now a global necessity. Despite the large number of courses available, the number of people who are seeking this kind of skill appears to be even larger. The business involved in this education is promising. Both established researchers and aspiring researchers need to pay attention to this trend.
